# The rs1142345 in TPMT Affects the Therapeutic Effect of Traditional Hypoglycemic Herbs in Prediabetes

**DOI:** 10.1155/2013/327629

**Published:** 2013-04-29

**Authors:** Xi Li, Feng-Mei Lian, Dong Guo, Lan Fan, Jie Tang, Jing-Bo Peng, Hong-Wen Deng, Zhao-Qian Liu, Xin-Hua Xiao, Yan-Rong Wang, Ke-Yi Qu, Sheng Deng, Qi Zhong, Yi-Ling Sha, Yan Zhu, Yu-Jing Bai, Xin-Yan Chen, Qiang Zhou, Hong-Hao Zhou, Xiao-Lin Tong, Wei Zhang

**Affiliations:** ^1^Pharmacogenetics Research Institute, Institute of Clinical Pharmacology, Central South University, Changsha, Hunan 410078, China; ^2^Guang'anmen Hospital, China Academy of Chinese Medical Sciences, Beijing 100053, China; ^3^Department of Endocrinology, Peking Union Medical College Hospital, China Academy of Medical Sciences, Beijing 100730, China; ^4^Department of Endocrinology, Peking University Third Hospital, Peking University, Beijing 100083, China; ^5^Department of Endocrinology, Yiling Hospital, Yichang, Hubei 443100, China; ^6^Department of Pharmacy, Xiangya Hospital, Central South University, Changsha, Hunan 410078, China; ^7^Department of Neurology, Xiangya Hospital, Central South University, Changsha, Hunan 410078, China; ^8^Department of Endocrinology, First People's Hospital of Hegang, Hegang, Heilongjiang 154100, China

## Abstract

Therapeutic interventions in prediabetes are important in the primary prevention of type 2 diabetes (T2D) and its chronic complications. However, little is known about the pharmacogenetic effect of traditional herbs on prediabetes treatment. A total of 194 impaired glucose tolerance (IGT) subjects were treated with traditional hypoglycemic herbs (Tianqi Jiangtang) for 12 months in this study. DNA samples were genotyped for 184 mutations in 34 genes involved in drug metabolism or transportation. Multinomial logistic regression analysis indicated that rs1142345 (A > G) in the thiopurine S-methyltransferase (TPMT) gene was significantly associated with the hypoglycemic effect of the drug (*P* = 0.001, FDR *P* = 0.043). The “G” allele frequencies of rs1142345 in the healthy (subjects reverted from IGT to normal glucose tolerance), maintenance (subjects still had IGT), and deterioration (subjects progressed from IGT to T2D) groups were 0.094, 0.214, and 0.542, respectively. Binary logistic regression analysis indicated that rs1142345 was also significantly associated with the hypoglycemic effect of the drug between the healthy and maintenance groups (*P* = 0.027, OR = 4.828) and between the healthy and deterioration groups (*P* = 0.001, OR = 7.811). Therefore, rs1142345 was associated with the clinical effect of traditional hypoglycemic herbs. Results also suggested that TPMT was probably involved in the pharmacological mechanisms of T2D.

## 1. Introduction 

Diabetes is a metabolic disorder characterized by chronic hyperglycemia and abnormalities in carbohydrate, fat, and protein metabolism. The World Health Organization (WHO) reported that diabetes is the fifth leading cause of death in the world and is a serious public health problem worldwide [1]. The prevalence of diabetes in the USA, European Union, China, and India is 8.3%, 6.5%, 9.7%, and 8.6%, respectively, and these numbers increase annually [2–5]. Approximately 366 million people are estimated to develop diabetes-related diseases by 2030 [6]. Type 2 diabetes (T2D) is the most common form of diabetes, accounting for over 90% of all cases.

Prediabetes is a condition in which the glycemic variables are lower than the diabetes threshold but higher than normal (the level of fasting plasma glucose (FPG) is lower than 6.1 mmol/L and the glucose level after a two-hour oral glucose tolerance test (2 h OGTT) is lower than 7.8 mmol/L). Prediabetes is of three types: impaired fasting glucose (IFG), impaired glucose tolerance (IGT), and combined IFG/IGT [7]. WHO (1999) defined IGT as a prediabetic state in which the level of FPG is lower than 7.0 mmol/L and the glucose level after a 2 h OGTT is between 7.8 mmol/L and 11.1 mmol/L. IFG is defined as a condition in which the level of FPG is between 6.1 mmol/L and 6.9 mmol/L and the 2 h OGTT glucose level is lower than 7.8 mmol/L. Previous study reported that 5% to 10% of prediabetics become diabetes patients every year; thus, prediabetes is a high-risk state for diabetes [8]. Hypoglycemic therapy significantly reduces the risk of developing diabetes [8, 9]. 

Individual variations in drug response are common in clinical treatments, which result in the low control rate of many diseases [10]. Many pharmacogenetic studies have been conducted to identify the potential mechanism of drug response differences, but only few focused on traditional herbs. Arrays have been developed mainly for pharmacogenetic studies [11–13]. Pharmacogenetic arrays include most of the markers related to drug absorption, distribution, metabolism, and excretion (ADME). Pharmacogenetic arrays help describe the relationship between the ADME markers and the drug under study. Consequently, this type of array has become popular in pharmacogenetic studies.

A traditional hypoglycemic herb known as Tianqi Jiangtang is widely utilized for diabetes treatment in China. The herb is processed and produced in capsules by Heilongjiang Baoquan Pharmaceutical Company, Limited (Hegang, China). This drug is licensed by the State Food and Drug Administration of China as a novel category III drug for lowering blood glucose levels in patients with diabetes [14, 15]. Previous studies have indicated that Tianqi Jiangtang controls diabetes by reducing hyperglycemia and modifying lipid metabolism [16, 17]. Tianqi Jiangtang consists of 10 Chinese herbal medicines, namely, Radix Astragali, Radix Trichosanthis, Fructus Ligustri Lucidi, Caulis Dendrobii, Radix Ginseng, Cortex Lycii Radicis bone, Rhizoma Coptidis, Asiatic Cornelian cherry fruit, Ecliptae Herba, and Chinese gall. Many of these herbal medicines are correlated with diabetes-related parameters. For example, Rhizoma Coptidis and astragalin in Radix Astragali reduce glucose, similar to Diformin [18, 19]. Berberine in Rhizoma Coptidis improves some glycemic parameters [20–22]. Ginsenoside Re in Radix Ginseng has significant antihyperglycemic effects [23–25]. The iridosides of Cornus officinalis in Asiatic Cornelian cherry fruit prevent diabetic vascular complications [26, 27]. Only one major effective component of Tianqi Jiangtang has been identified, namely, berberine hydrochloride (C_20_H_18_ClNO_4_), which has been successfully employed in antidiabetes treatments [28–31].

This study aims to identify a possible prophylactic action against diabetes as well as the genetic factors related to the individual differences in drug response to Tianqi Jiangtang. A total of 194 prediabetes patients from 12 Chinese hospitals were treated with traditional hypoglycemic herbs for 3 to 12 months. The condition of the subjects during the trial was determined, and a correlation analysis was conducted to identify the genetic markers related to the hypoglycemic effect of traditional hypoglycemic herbs.

## 2. Materials and Methods

### 2.1. Subjects

This study was approved by the Institutional Review Board of Beijing Guang'anmen Hospital, Chinese Academy of Medical Sciences, and by Central South University. All subjects were IGT patients from 12 hospitals in China. The hospitals are Beijing Guang'anmen Hospital, Chinese Academy of Medical Sciences; Zhejiang Chinese Medical University Affiliated Hospital; Kwong Hing/Hangzhou Municipal TCM Hospital; The First Affiliated Hospital of Guangzhou University of Traditional Chinese Medicine; Chinese Medicine Hospital of Foshan City, Guangdong Province; Qinghai Hospital of Traditional Chinese Medicine, Qinghai Province; Shenzhen Traditional Chinese Hospital; Affiliated Hospital of Changchun University of Traditional Chinese Medicine; Chinese Medicine Hospital of Xuzhou; Chinese Medicine Hospital of Shantou; Guangzhou Huangpu Chinese Medicine Hospital; and Guangzhou Tianhe Chinese Medicine Hospital. All subjects were overweight or obese. The subjects signed an informed consent form before participating in this study. The inclusion and exclusion criteria are provided below.

Inclusion criteria are (1) having an FPG level lower than 7.0 mmol/L and a glucose level between 7.8 mmol/L and 11.1 mmol/L after a two-hour OGTT; (2) being Qi- and yin deficient according to traditional Chinese medicine and suffering from excess body heat (manifested by weakness, fatigue, dry mouth, bitter taste in mouth, red tongue with teeth prints, thin and white hair, and taut, rapid, or thready pulse); (3) having never used an anti-diabetic drug; (4) having body mass index (BMI) of 24 kg/m^2^ to 30 kg/m^2^; (5) aging 25 to 70; (6) not participating in drug trials in the past three months.

Exclusion criteria are (1) having suffered from acute cardiocerebrovascular events or myocardial infarction in the past six months; (2) having proliferative retinopathy treated by laser; (3) having stress or secondary hyperglycemia; (4) being unwilling to cooperate; (5) having mental illness; (6) being pregnant or lactating, or planning pregnancy or lactation; (7) being allergic to Tianqi Jiangtang; (8) having systolic blood pressure higher than or equal to 160 mmHg and diastolic blood pressure higher than or equal to 100 mmHg (i.e., secondary hypertension); (9) having cholesterol higher than or equal to 6.22 mmol/L or low-density lipoprotein higher than or equal to 4.14 mmol/L; (10) taking other hypoglycemic drugs.

### 2.2. Herbs and Preparation Method

Tianqi Jiangtang capsules are produced by Heilongjiang Baoquan Pharmaceutical Company, Limited (Hegang, China; Z20063799). The exact amount of each of the herbs in the drug is listed in Table 1. The preparation of the drug was described in detail by Zhang et al. [16]. The batch number of the drug is 080103. The berberine hydrochloride content of each capsule was measured by high-performance liquid chromatography to control drug quality. Exactly 0.5 g of the sample was added to a conical flask containing 50 mL of acidified (1% HCl) methanol. The mixture was heated ultrasonically (250 W, 40 kHz) for 30 min and then naturally cooled and filtered. Ten milliliters of the filtered mixture was placed in a neutral alumina column (100 mesh to 200 mesh, 5 g, inner diameter of 0.9 cm). Ethanol (30 mL) was utilized to elute the mixture. The eluent was collected and transferred to a 50 mL measuring flask. Ten microliters of the eluent was injected into the liquid chromatograph (Beckman Instruments, Inc.) to determine berberine hydrochloride content. The average berberine hydrochloride content was 7.8 (±0.13) mg per capsule.

### 2.3. Study Design

**Figure 1:**
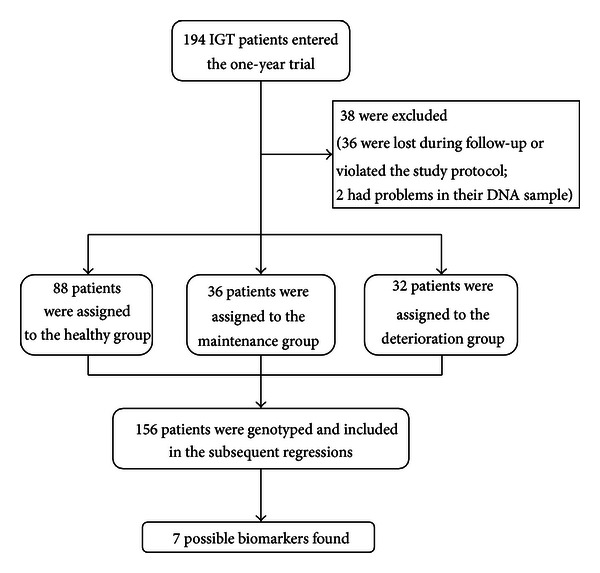
Research process.

This study is a double-blind, multicenter, randomized clinical trial (registration number: ISRCTN90063632). The 194 volunteers participated in a one-month placebo treatment before the actual treatment. Afterward, the subjects were treated with Tianqi Jiangtang at a dosage of 5 capsules (1.6 g, based on the instruction of the drug) per time, three times daily for 3 months to 12 months. Smoking and drinking alcohol were prohibited during the treatment. The subjects were subjected to blood glucose tests every three months and were divided into healthy, maintenance, and deterioration groups according to the test results. The WHO criteria were employed to diagnose and classify the samples [32]. The healthy group included subjects whose FPG and 2 h OGTT levels returned to normal (the FPG level was less than 6.1 mmol/L and the glucose level after the 2 h OGTT was less than 7.8 mmol/L). This group then underwent a consolidation period of 3 months, taking the medicine at a dosage of 3 capsules per time, three times daily. Treatment was stopped after this period, but the group was monitored for six months. The maintenance group included subjects who still had IGT (the FPG level was less than 7.0 mmol/L and the glucose level after the 2 h OGTT was between 7.8 mmol/L and 11.1 mmol/L) after the trial. This group received treatment until the end of the study. Subjects who developed T2D (the FPG level was more than or equal to 7.0 mmol/L or the glucose level after the 2 h OGTT was more than or equal to 11.1 mmol/L) during the trial were assigned to the deterioration group. The treatment for this group was substituted with Western medicine. The research process is illustrated in Figure 1.

### 2.4. Genotyping

A commercial isolation kit (Qiagen, Inc.) was utilized to extract genomic DNA from peripheral blood leukocytes. DNA concentration and purity were assessed with Nanodrop 2000 (Thermo Fisher Scientific, Inc.). An Illumina VeraCode ADME core panel (Illumina, Inc.) was utilized according to the manufacturer's protocol to genotype the subjects. With this core panel, the genotypes of the 32 samples were recorded. The DNA of each sample was divided into three portions and the target regions into three optimized assay pools by utilizing three different biotinylated primer mixes. Paramagnetic particles were employed to isolate the products. The allele-specific extension and ligation of the products were conducted by universal polymerase chain reaction amplification. The products were then hybridized with VeraCode beads and scanned by a BeadXpress Reader (Illumina, Inc.). The data were managed and analyzed through Verascan. The VeraCode ADME core panel is described in detail at http://www.illumina.com/documents/products/datasheets/datasheet_veracode_adme_core_panel.pdf. The VeraCode ADME core panel includes 184 markers (173 single-nucleotide polymorphisms (SNPs), 10 copy number variations, and 1 three-base deletion) in 34 genes, covering over 95% of the PharmaADME Core List. All genes were functionally significant in drug ADME (Table 2). SNPs with a minor allele frequency (MAF) lower than 5%, a Hardy-Weinberg equilibrium (HWE) test *P* value lower than 0.001, and those that were not genotyped were excluded before the statistical analyses. Out of the initial full set of 173 SNPs, 3 were not genotyped, 127 had MAF lower than 0.05, and none had an HWE test *P* value lower than 0.001. The 43 SNPs that remained were employed for subsequent analysis.

### 2.5. Statistical Analyses

Stepwise regression was performed (add *P* = 0.05, remove *P* = 0.1) before the correlation analysis to screen for possible covariants. The analysis parameters were age, sex, height, weight, body mass index, systolic and diastolic blood pressure, heart rate, and waistline and hip measurements. Multinomial and binary logistic regressions were performed with SPSS 17 (Chicago, IL) to identify the association between the genetic markers and the hypoglycemic effects of Tianqi Jiangtang. The Bonferroni correction was utilized for the multiple testing corrections to adjust the raw *P* values. Plink 1.07 (http://pngu.mgh.harvard.edu/~purcell/plink/) was utilized to calculate the MAFs and HWE test *P* values of the SNPs. 

## 3. Results

The basic characteristics of the subjects including sex, age, height, weight, BMI, and FPG and 2 h OGTT levels are summarized in Table 3. Among the initial 194 prediabetes patients, the 156 patients who finished the entire treatment were selected for subsequent analysis. The average FPG and 2 h OGTT glucose levels were 6.15 ± 0.54 mmol/L and 9.22 ± 1.03 mmol/L, respectively. Among the selected 156 patients, 88 were cured (healthy group), 36 still had IGT (maintenance group), and 32 developed T2D (deterioration group). The efficiency of the Tianqi Jiangtang capsules was 56%. The healthy group included 46 males and 42 females aged 29 to 67. The average FPG and 2 h OGTT glucose levels of this group were 5.64 ± 0.59 mmol/L and 6.86 ± 1.12 mmol/L, respectively. The corresponding average decrease was 0.40 ± 0.81 mmol/L for average FPG and 2.13 ± 1.41 mmol/L for 2 h OGTT glucose. The maintenance group consisted of 17 males and 19 females aged 32 to 69. The average FPG and 2 h OGTT glucose levels were 6.06 ± 0.58 mmol/L and 9.01 ± 1.56 mmol/L, respectively. The deterioration group included 15 males and 17 females aged 32 to 69. The average FPG and 2 h OGTT glucose levels were 7.34 ± 1.08 mmol/L and 11.00 ± 1.76 mmol/L, respectively. The corresponding average increase was 1.03 ± 1.17 mmol/L for FPG and 1.41 ± 1.86 mmol/L for 2 h OGTT glucose.

**Figure 2:**
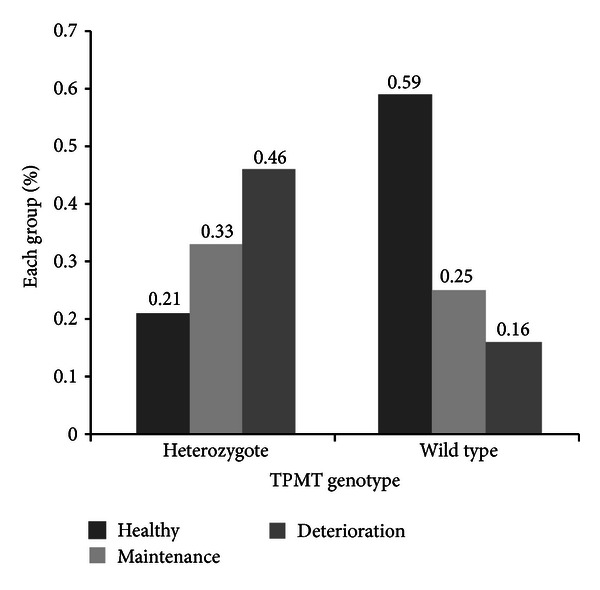
Percentage of each group in rs1142345 heterozygote (AG) and wild-type (AA) patients.

Age (*P* = 0.032) was screened as the covariate of subsequent multinomial logistic regressions. Seven SNPs were found to be significantly associated with the hypoglycemic effect of Tianqi Jiangtang after the multinomial logistic regressions. These SNPs were rs1142345 (*P* = 0.001) in the TPMT (thiopurine S-methyltransferase) gene, rs2306168 (*P* = 0.003) in the ABCG2 (adenosine triphosphate ATP-binding cassette, subfamily G, member 2) gene, rs2231142 (*P* = 0.021) in the SLCO2B1 (solute carrier organic anion transporter family, member 2B1) gene, rs717620 (*P* = 0.023) in the ABCC2 (ATP-binding cassette, sub-family C, member 2) gene, rs1799931 (*P* = 0.024) in the NAT2 (N-acetyltransferase 2) gene, rs4244285 (*P* = 0.027) in the CYP2C19 (cytochrome P450, family 2, sub-family C, polypeptide 19) gene, and rs4124874 (*P* = 0.044) in the UGT1A1 (uridine diphosphate glucuronosyltransferase 1 family, polypeptide A1) gene. Only rs1142345 in TPMT passed the multiple testing correction (adjust *P* = 0.043). The analysis results and the basic characteristics of the seven SNPs are summarized in Table 4. The “G” allele frequencies of rs1142345 in the healthy, maintenance, and deterioration groups were 0.094, 0.214, and 0.542, respectively. These frequency values increased progressively.  Figure 2 shows the percentage of each group in the heterozygote (AG) carriers (TPMT rs1142345 mutated) and homozygote (AA) carriers (wild-type allele). No mutated homozygotes (GG) of TPMT rs1142345 were found in the subjects. The effective ratio of subjects with the wild-type allele of TPMT rs1142345 was 2.8 times higher than that of subjects with TPMT heterozygotes. Binary logistic regression was also employed. Between the healthy and maintenance groups and between the healthy and deterioration groups, rs1142345 was significantly associated with the hypoglycemic effect of the drug (*P* = 0.027, OR = 4.828 and *P* = 0.001, OR = 7.811, respectively).

## 4. Discussion 

To our knowledge, this study is the first to utilize the ADME gene chip in the pharmacogenetic study of traditional hypoglycemic herbs. The seven SNPs were associated with the hypoglycemic effect of Tianqi Jiangtang. The genes containing these SNPs are all pharmacokinetics related, including phase I enzyme CYP2C19; phase II enzymes TPMT, NAT2, and UGT1A1; transporter ABCC2, SLCO2B1, and ABCG2. Only rs1142345 in TPMT passed the multiple testing corrections. 

TPMT catalyzes the S-methylation of some drugs, such as azathioprine, mercaptopurine, and thioguanine. Research on TPMT genetic polymorphisms is one of the most advanced pharmacogenetic studies [33]. The TPMT activity exhibits genetic polymorphism: approximately 90% of individuals inherit high activity, 10% exhibit intermediate activity because of heterozygosis, and approximately 0.3% have low or no detectable enzyme activity because they inherited two nonfunctional TPMT alleles [34]. The SNP rs1142345, also called TPMT*3B, alters Ala154Thr and reduces the TPMT activity. In this study, TPMT*3B was associated with the hypoglycemic effect of Tianqi Jiangtang. The effective ratio of subjects with homozygotes (AA) of the wild-type allele of TPMT rs1142345 was 2.8 times higher than that of subjects with TPMT heterozygotes (AG). This association is due to the possible involvement of TPMT with one of the active components of Tianqi Jiangtang. Reduced TPMT activity increases the concentration of the active components and improves their pharmacological effect. However, the relationship between TPMT and berberine hydrochloride (the major effective active component of Tianqi Jiangtang) remains unclear. The hypoglycemic effect on subgroup patients with a TPMT mutated allele and berberine hydrochloride from Tianqi Jiangtang should be investigated further.

Although the other six polymorphisms did not pass the multiple testing corrections, they still provide information for further study. The polymorphism rs717620 (−24C>T) in ABCC2 was found to be associated with the hypoglycemic effect of Tianqi Jiangtang. ABCC2 encodes the excretive transporter MRP2, mediating the reversed concentration gradient excretion of some exogenous and endogenous compounds. The polymorphism rs717620 enhances transport activity and substrate excretion [35, 36]. Subjects with a mutated allele (T) of rs717620 tended to progress from IGT to diabetes mellitus (the “T” allele frequency of the healthy group was 0.35 and that of the deterioration group was 0.52). The enhanced excretion of the active components of mediated reversed concentration gradient excretion decreased cell concentration and worsened the pharmacological reaction. The polymorphism rs2231142 (421C>A) in the excretive transporter ABCG2 was associated with the suitable hypoglycemic effect of mediated reversed concentration gradient excretion because rs2231142 possibly weakened the transport function and increased the plasma concentration of its substrate [37]. One polymorphism in the uptake transporter gene associated with the hypoglycemic effect of Tianqi Jiangtang was rs2306168 (1457C>T) in SLCO2B1 (encoded OATP2B1). This polymorphism reduced transport activity [38, 39] and improved the pharmacological reaction. OCT1 is an organic cation transporter, whereas OATP2B1 is an anion transporter. This difference suggests that Tianqi Jiangtang has more than one kind of active compound. The polymorphisms rs4244285 (682C>A) in CYP2C19, rs1799931 (857G>A) in NAT2, and rs4124874 (−3279T>G) in UGT1A1 were also associated with the hypoglycemic effect of Tianqi Jiangtang. The subjects with the mutated alleles of these three polymorphisms exhibited reduced catalytic activity [40–42] and appropriate pharmacological reactions. Although these six polymorphisms did not pass the multiple testing corrections, their results may provide clues for the screening of possible active components.

The rate of prediabetes developing into diabetes was 20.5% in this study, a value that is higher than that reported in a previous study (5% to 10%) [8]. The higher rate could have been caused by the difference in the design of the studies. The subjects in this present study were selected from the Chinese Han population, whereas the previous study focused on Caucasians. The ethnic differences between the Chinese and Caucasian populations may have caused the rate to vary. The small sample size of the present study may have also caused the variation. Moreover, the traditional hypoglycemic herbs administered in this study might have been unsuitable for the IGT patients because of their genetic background. For these patients, taking traditional hypoglycemic herbs increased the risk of diabetes, which could have caused the rate to increase.

## 5. Conclusion

The SNP rs1142345 was found to be associated with the clinical consequence of Tianqi Jiangtang. TPMT is possibly involved in the pharmacological mechanisms of T2D. 

## Figures and Tables

**Table 1:** Exact amount of each component required to produce 1000 capsules of Tianqi Jiangtang.

Herb name	Amount	Herb name	Amount
Radix Astragali	400 g	Radix Trichosanthis	400 g
Fructus Ligustri Lucidi	334 g	Caulis Dendrobii	200 g
Radix Ginseng	134 g	Cortex Lycii Radicis bone	267 g
Rhizoma Coptidis (steamed)	200 g	Asiatic Cornelian cherry fruit	200 g
Ecliptae Herba	334 g	Chinese gall	200 g

**Table 2:** Genes in the Veracode ADME core panel.

Phase I enzymes		Phase II enzymes		Transporters		Other
CYP1A1	CYP2C9	DPYD	SULT1A1	ABCB1	SLC22A6	VKORC1
CYP1A2	CYP2D6	GSTM1	TPMT	ABCC2	SLCO1B1	
CYP2A6	CYP2E1	GSTP1	UGT1A1	ABCG2	SLCO1B3	
CYP2B6	CYP3A4	GSTT1	UGT2B15	SLC15A2	SLCO1B1	
CYP2C19	CYP3A5	NAT1	UGT2B17	SLC22A1		
CYP2C8		NAT2	UGT2B7	SLC22A2		

**Table 3:** Basic characteristics of the subjects in the study.

Traits	Total sample	Healthy group	Maintenance group	Deterioration group	*P* value
Number (male/female)	156 (78/78)	88 (46/42)	36 (17/19)	32 (15/17)	—
Age (years)	51.96 ± 9.78	50.59 ± 9.49	52.83 ± 9.22	54.7 ± 10.74	0.10
Height (cm)	163.20 ± 8.21	162.74 ± 8.49	163.25 ± 7.63	164.41 ± 8.18	0.62
Weight (kg)	66.65 ± 9.79	66.60 ± 10.42	66.21 ± 6.60	67.31 ± 11.16	0.90
BMI (kg/m^2^)	24.99 ± 3.12	25.06 ± 2.99	24.90 ± 2.76	24.91 ± 3.84	0.96
FPG (mmol/L)	6.15 ± 0.54	5.64 ± 0.59	6.06 ± 0.58	7.34 ± 1.08	0.02
2 h OGTT (mmol/L)	9.22 ± 1.03	6.86 ± 1.12	9.01 ± 1.56	11.00 ± 1.76	0.01

The values are presented as means ± standard deviation.

The *P* values in all three groups were calculated by ANOVA.

**Table 4:** Significant SNPs for multinomial logistic regression.

SNP	Gene	Allele	Chr	Position	MAF*	*P *	FDR *p *
rs1142345	TPMT	A/G	6	18130918	0.229	0.001	0.043
rs2306168	SLCO2B1	T/C	11	74907582	0.305	0.003	0.129
rs2231142	ABCG2	A/C	4	89052323	0.260	0.021	0.903
rs717620	ABCC2	C/T	10	101542578	0.230	0.023	0.989
rs1799931	NAT2	A/G	8	18258370	0.153	0.024	1
rs4244285	CYP2C19	A/G	10	96541616	0.344	0.027	1
rs4124874	UGT1A1	G/T	2	234665659	0.337	0.044	1

*Minor allele frequency calculated from the subjects.
